# *Humulus scandens*-Derived Biochars for the Effective Removal of Heavy Metal Ions: Isotherm/Kinetic Study, Column Adsorption and Mechanism Investigation

**DOI:** 10.3390/nano11123255

**Published:** 2021-11-30

**Authors:** Xingang Bai, Luyang Xing, Ning Liu, Nana Ma, Kexin Huang, Dapeng Wu, Mengmeng Yin, Kai Jiang

**Affiliations:** 1 Key Laboratory for Yellow River and Huai River Water Environmental and Pollution Control, Henan Key Laboratory for Environmental Pollution Control, Ministry of Education, School of Environment, Henan Normal University, Xinxiang 453007, China; baixghtu@126.com (X.B.); ke1919124086@163.com (K.H.); ymmdyx0125@163.com (M.Y.); 2 Key Laboratory of Green Chemistry Medias and Reactions, Ministry of Education, School of Chemistry and Chemical Engineering, Henan Normal University, Xinxiang 453007, China; xingluyang0517@163.com (L.X.); mann076@htu.edu.cn (N.M.)

**Keywords:** biochar, adsorption, molten salt, heavy metal ion, water treatment

## Abstract

*Humulus scandens* was first adopted as a biomass precursor to prepare biochars by means of a facile molten salt method. The optimized biochar exhibits a high specific surface area of ~450 m^2^/g, a rich porous structure and abundant oxygen functional groups, which demonstrate excellent adsorption performance for heavy metal ions. The isotherm curves fit well with the Langmuir models, indicating that the process is governed by the chemical adsorption, and that the maximum adsorption capacity can reach 748 and 221 mg/g for Pb^2+^ and Cu^2+^, respectively. In addition, the optimized biochar demonstrates good anti-interference ability and outstanding removal efficiency for Cu^2+^ and Pb^2+^ in simulated wastewater. The mechanism investigation and DFT calculation suggest that the oxygen functional groups play dominant roles in the adsorption process by enhancing the binding energy towards the heavy metal ions. Meanwhile, ion exchange also serves as the main reason for the effective removal.

## 1. Introduction

With the rapid development of China’s economy, a large volume of wastewater containing heavy metal ions is being discharged by the mining, metallurgy, chemical, electronics and other production industries [1,2]. Heavy metal ion pollution remaining in different water sources seriously threatens people’s safety and health [3,4]. Harmful heavy metal ions such as cadmium, chromium, lead, arsenic, copper, mercury and so on are commonly detected in different water bodies [5,6]. At present, chemical, physical and biological methods including electrochemical treatment, ion exchange, chemical precipitation and adsorption are the mostly commonly employed strategies to remove heavy metal ions from wastewater [7]. Among these methods, adsorption is considered an effective and economic method because of its facile operation and low cost [8,9]. At present, biomass-derived activated carbons with a large specific surface area and resource abundance have been widely used in wastewater treatment [10,11,12]. A large number of studies have proven that the outstanding adsorption performance of biochar originates from the large surface area, stable porous structure, abundant surface oxygen functional groups such as carboxyl and hydroxyl groups, and rich mineral species which can remove the harmful ions through the ion exchange process [13]. Therefore, it is of great significance to develop high-efficiency and low-cost biochar materials to remove heavy metals from polluted water environments [14].

Hitherto, many biomasses, such as agricultural wastes, woody materials, algae and so on [15,16,17,18], are widely employed to prepare biochar for the removal of heavy metal ions from the water system. For example, *Undaria* was adopted as a raw material to prepare biochar through rapid pyrolysis and physical steam activation. The as-obtained biochar was able to effectively remove Cu^2+^ from the aqueous solution [19]. In order to achieve the fast removal of Pb^2+^, biomasses such as palm, soybean meal, shells and so on were also adopted to prepare biochars with more adsorption sites as well as rich ion diffusion channels [20,21,22,23,24]. Based on these studies, it was found that although they are prepared through similar treatment methods, the adsorption performance of biochars varies widely, which implies that the unique tissue structures as well as the composition of the biomass exerts great influence on the adsorption performance of the biochar. Therefore, it is of great significance to explore the family of biomass for high-performance biochars.

In addition, in order to improve the adsorption capacity of biochars, various approaches have been developed to achieve the chemical modification of amino, alkali, sulfur and phosphoric function groups on the biochar surface. For example, Zhang et al. modified the surface of a rice straw-derived biochar with an amino group through nitrification and amination to prepare high-efficiency Pb^2+^ adsorbent. The as-introduced amino group shows strong complexation with Pb^2+^ to improve the adsorption strength of biochars [23]. Ming et al. adopted EDTA to modify the activated carbon derived from shells, and the mechanism studies indicate that the electrostatic action and complexation of surface active groups play important roles in enhancing the Pb^2+^ adsorption capacity [24]. Zhu et al. prepared surface-oxidized biochar from porous biochar with a high Cd^2+^-adsorption capacity, and they found that the adsorption capacity is mainly affected by the surface functional groups [25]. In addition to the oxygen and amino function groups, Cl active sites and sulfur groups were also deliberately introduced on the surface of the biochars to enhance the removal efficiency of Hg^+^ and Cu^2+^, taking advantage of the enhanced binding energy between the active sites and the target heavy metal ions [26,27]. Although many schemes have been developed to achieve the surface modification of biochars, these strategies still suffer from many drawbacks, such as a tedious preparation procedure, high cost, toxic modification reagents and potential secondary contamination risk. In addition to the modification, the current production of biochars usually requires inert gas protection and high-temperature treatment with corrosive activators, which often have many disadvantages such as a high cost and a complicated preparation process. Therefore, it is necessary to develop a facile preparation process to produce biochar with rich surface function groups through a one-step pyrolysis process.

The molten salt method represents a commonly adopted route to prepare porous carbon materials with inert salt as the sealing and activating bi-functional medium. The biochars prepared through the molten salt method are widely used in the fields of environmental adsorption, energy storage devices, air purification and so on [28]. Due to the etching effect of molten salt and the template effect of salt crystals, mesopores and macropores can be introduced into the products [29]. At the same time, oxygen in the atmosphere can further react with highly active carbon atoms, which could introduce a large number of micropores into the products [30]. Therefore, the biochar prepared by the molten salt method has a high specific surface area and rich oxygen functional groups, which is conducive to the adsorption performance [31].

*Humulus scandens* (HS) is a perennial climbing herb which is widely distributed in the northern region of China. However, the massive growth of HS causes negative impacts on agricultural production and human activities. Moreover, the tissues of HS have regular porous structure and rich cellulose, which allows it to be promising precursor to prepare porous carbon materials. In this study, HS was firstly used as a precursor to prepare biochar by means of the NaOH modified molten salt method. The removal efficiency of Cu^2+^ and Pb^2+^ ions from an aqueous solution was greatly enhanced due to the well-developed porous structure, rich oxygen function groups as well as the embedded mineral species. The maximum Q_m_ can reach 748 and 221 mg/g for Pb^2+^ and Cu^2+^, respectively. In addition, the effects of adsorption conditions including initial pH, contact time, and initial concentration on the adsorption capacities were systematically investigated. The adsorption mechanism was studied, which indicates that oxygen function groups and the imbedded mineral species play dominant roles in the adsorption process by enhancing the binding energy towards the heavy metal ions and facilitating the ion exchange process. In addition, the optimized biochar shows outstanding anti-interference ability and good column adsorption performance, permitting its potential application in industrial-scale utilization. Considering that HS is a harmful biomass for agricultural production, the effective conversion of HS into biochar with excellent adsorption capacity can not only enrich the biomass family to produce carbon materials, but also reduce the adverse effects of the massive growth of HS in the north of China.

## 2. Materials and Methods

### 2.1. Materials

*Humulus scandens*, collected at Henan Normal University in Xinxiang City (35°19′54.49″ N, 113°54′51.75″ E) (Supplementary Materials Figure S1), Henan Province, China, was washed and dried in an oven at 50 °C for 12 h. The dried and pulverized HS was screened through a 100-mesh sieve for later use. Pb(NO_3_)_2_, Cu(NO_3_)_2_·5H_2_O, NaOH, NaCl and KCl were purchased from Sinopharm Chemical Reagent Co. Ltd. (Beijing, China), were of analytic grade and were used without further purification.

### 2.2. Preparation of the Biochar

NaCl and KCl were mixed in an agate mortar at a mass ratio of 1:1. Then, 3 g of HS powder was mixed with 6 g of mixed salt and placed in a 50 mL ceramic crucible, covered with about 1 cm of mixed salt. The mixture was heated to 400, 500, 600, 700 and 800 °C in a muffle furnace at a rate of 10 °C/min, respectively. After continuous pyrolysis for 3 h, it was naturally cooled and cleaned with DI water to neutral. After drying in a 60 °C vacuum oven for 12 h, a series of biochars were obtained, and the biochar material obtained at a pyrolysis temperature of 600 °C was named HSC-M.

Then, 1, 2 and 3 g of NaOH were added to the mixture of 3 g HS powder and 6 g salt. After grinding evenly, the mixture was transferred into a ceramic pot, and covered with salt mixture with a thickness of about 1 cm. It was heated in a muffle oven at a rate of 10 °C/min to 600 °C and maintained for 3 h. The as-prepared biochars were named HSC-MA-1, HSC-MA-2 and HSC-MA-3 according to the NaOH dosage.

In addition, the washed, dried and crushed HS powder was placed in a ceramic crucible and placed in a tubular furnace. Under N_2_ protection, the powder was sintered for 3 h at 600 °C with a heating rate of 10 °C/min. After having cooled naturally, the biochar was demoted as HSC-N_2_.

### 2.3. Characterization of the Biochar

The pyrolysis process of the biomass in the molten salt medium was studied using TG-DSC in the air. The surface morphology and element distribution of biochar were analyzed by means of field emission scanning electron microscopy equipped with an energy dispersion spectrometer (SEM, Hitachi SU8010, Tokyo, Japan). The internal structure of the samples was observed using transmission electron microscopy (TEM, TF20, JEOL 2100F, Tokyo, Japan). X-ray photoelectron spectroscopy (XPS, Escalab250XI, Thermo Fisher Scientific, Waltham, MA, USA) was used to record the surface elemental composition and chemical bonds of the samples. X-ray powder diffraction (XRD, Rigaku DMAX-2000, Rigaku, Tokyo, Japan) was used to demonstrate the lattice space structure of the sample. The contents of Ca, Mg, Fe and Cu in the samples were analyzed by inductively coupled plasma mass spectrometry (ICP-MS, ELAN DRC-e, PerkinElmer Inc, LAS, USA). A Fourier transform infrared spectrometer (FTIR, Nexus 470, GMI Inc., Lebanon, OH, USA) and Raman spectrometer (Labramhr EVO, Horiba France SAS, Palaiseau, France) were used to analyze the surface functional groups of the biochar in the wave number ranges of 4000~400 cm^−1^ and 3000~50 cm^−1^, respectively. The specific surface area and pore diameter distribution of the samples were determined by means of the N_2_ adsorption method using a surface area analyzer (BET, MicrotracBEL, Osaka, Japan).

### 2.4. Heavy Metal Sorption Experiments

#### 2.4.1. Adsorption Experiment

Cu(NO_3_)_2_·5H_2_O and Pb(NO_3_)_2_ were added into two 500 mL volumetric flasks, respectively, and were prepared with 1000 mg/L Cu^2+^ and Pb^2+^ reserve solutions. Ten milligrams of the as-prepared samples were added into a 250 mL conical flask containing 100 mL, 100 mg/L Pb^2+^ or Cu^2+^, respectively. The suspension was oscillated in a constant temperature water bath oscillator at 180 r/min for 4 h at 25 °C. After the reaction, the suspension was filtered using a 0.45 μm cellulose acetate membrane. Finally, the concentration of Pb^2+^ and Cu^2+^ after adsorption equilibrium was determined using a flame atomic absorption spectrometer.

#### 2.4.2. Influence of Initial pH Value

The initial pH value of 100 mg/L Cu^2+^ and Pb^2+^ solution was adjusted to 2, 3, 4, and 5 with 0.1 mol/L HNO_3_ or 0.1 mol/L NaOH solution. The effect of pH value on the adsorption of Pb^2+^ and Cu^2+^ by biochar in 100 mL of the Pb^2+^ and Cu^2+^ solution was determined by using the above method with a HSC-MA-2 dosage of 10 mg and a concentration of 100 mg/L.

#### 2.4.3. Influence of Adsorbent Dosage

To analyze the influence of adsorbent dosage, 10, 20, 50, 80, 100, and 200 mg of the biochar HSC-MA-2 were added into a conical flask containing 100 mL of 200 mg/L Pb^2+^. Similarly, 10, 20, 50, 80, 100 and 200 mg of the biochar HSC-MA-2 were also introduced into a conical flask containing 100 mL of 100 mg/L Cu^2+^. Different adsorbent concentrations (0.1–2.0 g/L) were studied to determine their influence on the removal of Pb^2+^ and Cu^2+^ ions.

#### 2.4.4. Adsorption Kinetics and Isothermal

For the kinetics tests, the initial concentration of Pb^2+^ was set as 200 mg/L and the biochar amount was set as 0.2 g/L. Meanwhile, the initial solubility of Cu^2+^ was 100 mg/L, and the amount of biochar was 0.5 g/L. The contact time between the biochars and the metal ion solution was 5–300 min, and the adsorption capacities of Pb^2+^ and Cu^2+^ on biochar were measured at 5, 10, 20, 30, 60, 120, 180, 240 and 300 min, respectively.

For the isothermal tests, the initial concentration of Pb^2+^ was set as 20, 50, 100, 150, 200, 250, and 300 mg/L, and the addition amount of biochar was 0.2 g L^−1^, and the initial concentration of Cu^2+^ was 20, 40, 80, 120, 160, 200, 240 mg/L, and the addition amount of biochar was 0.5 g/L. At a constant temperature of 25 °C, the water bath was oscillated at a speed of 180 RPM for 4 h, and the samples were sampled at adsorption equilibrium. After filtration, the concentrations of Pb^2+^ and Cu^2+^ in the filtrate were determined, and the adsorption capacities of the biochar HSC-MA-2 on Pb^2+^ and Cu^2+^ at corresponding initial concentrations were calculated.

The amount of heavy metal ions adsorbed per unit mass of the adsorbent ($Q_{e}$) was calculated from Equation (1):

(1) Qe=(c0−ce)×VW

The formula for calculating the removal rate of metal ions is:

(2) $$\eta \%=\frac{c_{0}-c_{e}}{c_{0}}\times 100\%$$

$c_{0}$ is the initial concentration of the Cu^2+^ and Pb^2+^ ions (mg L^−1^); $c_{e}$ is the concentration of the Cu^2+^ and Pb^2+^ ions at adsorption equilibrium (mg L^−1^);$\;Q_{e}$ is the amount of heavy metal ions adsorbed per unit mass of the adsorbent (mg g^−1^); V is the total volume of the solution (L); and W is the mass of the biochar (g).

In order to study adsorption mechanism and kinetic parameters, quasi-first order, quasi-second order and intra-molecular diffusion models were employed to fit the adsorption curves, respectively.

Quasi first order dynamic equation:

(3) $$\ln\left(q_{e}-q_{t}\right)=\ln q_{e}-\frac{k_{1}}{2.303}t$$

Quasi second order dynamic equation:

$$\frac{t}{q_{t}}=\frac{1}{k_{2}q_{e}^{2}}+\frac{t}{q_{e}}$$

The equation of the intramedullary diffusion model:

$$q_{t}=K_{\mathrm{id}}t^{1/2}$$

In the equation, $q_{e}\;\mathrm{and}\;q_{t}$ are the adsorption capacity at adsorption equilibrium and the adsorption capacity at time *t*, respectively (mg/g); *t* is the adsorption time (min); $k_{1}$ is the primary kinetic adsorption rate constant (min^−1^), and $k_{2}$ is the secondary kinetic adsorption constant (g (mg/min)^−1^). The correlation coefficient obtained by the fitting calculation was used to determine the adsorption process.

Adsorption isotherms of adsorbents describe the relationship between solid and liquid phases. Langmuir and Freundlich models are two commonly used adsorption isotherm models. Langmuir and Freundlich adsorption isotherm models are defined as follows:

Freundlich equation:

$$\ln q_{e}=\frac{1}{n}\ln C+\ln k_{f}$$

Langmuir equation:

$$\frac{C}{q_{e}}=\frac{1}{q_{m}k_{l}}+\frac{C}{q_{e}}$$

In the equation, C is the adsorption concentration (mg/L),$\;q_{e}$ is the equilibrium adsorption capacity (mg/g), $q_{m}$ is the maximum adsorption capacity (mg/g),$\;k_{l}$ is the Langmuir constant (L/mg), $k_{f}$ is the characteristic constant proportional to the equilibrium constant (L/mg), and n is the characteristic constant representing the properties of adsorption force.

### 2.5. The Effect of Background Ions

The effect of background ions on the adsorption of Pb^2+^ and Cu^2+^ ions by biochar was studied; 10 and 20 mg of HSC-MA-2 were added to 100 mL of 100 mg/L Pb^2+^ and 100 mg/L Cu^2+^ containing sodium, magnesium and calcium ions (with concentration of 1, 3, 5, 7 and 10 mmol/L), respectively, and then oscillated for 4 h at pH 5.0 and 25 °C.

### 2.6. Multi-Metal Adsorption

The interaction of coexisting heavy metals in the adsorption process has more practical significance than the interaction of a single system. A mixture of Pb^2+^, Cu^2+^, and Cr^3+^ was prepared at pH 5.0, and the initial concentrations of all metal ions were set as 50, 100, 150 and 200 mg/L. Different doses (0.5–3.0 g/L) of HSC-MS-2 were added into 100 mL of mixed heavy metal solution and reacted at 25 °C for 4 h.

### 2.7. Column Sorption

The actual wastewater simulation was estimated by the column sorption trials. The column adsorptions were carried out in a glass column (11.0 mm in diameter and 150 mm in length). The bottom of column was firstly packed with quartz sand (100 meshes). Then, 1.0 g of HSC-MA-2 was inserted on top of the quartz sand layer. The actual wastewater was pumped into the BC-ST packing bed in an up-flow type. The flow rate (Q, mL/min) of effluents was controlled by a peristaltic pump.

The simulated wastewater was fetched and prepared from the Yellow River in Xinxiang city, Henan province (34°54′17.78″ N, 113°46′7.95″ E) (Supplementary Materials Figure S2). The Cu^2+^, Pb^2+^ and Cr^3+^ concentration were controlled at 100 mg/L with a pH of 5 to determine the effect of wastewater treatment on column sorption.

### 2.8. Gaussian Computations

All computations were performed by the Gaussian 09 Program (Revision D.01, Gaussian, Inc., Wallingford, CT, USA). All the geometries of the carbon domains with different functional groups were optimized at the B3LYP/6-31+G(d) level. The electrostatic potential (ESP) maps describe the electron density distribution, and the red area represents electron-rich areas, while the green area represents electron-deficient areas. According to the ESP maps, the geometries were further optimized at the B3LYP/6-31+G(d)/SDD level. The interaction energy between M and xxx were calculated by considering basis set superposition error (BSSE) correction.

## 3. Results and Discussion

### 3.1. Material Characterization

**Figure 1:**
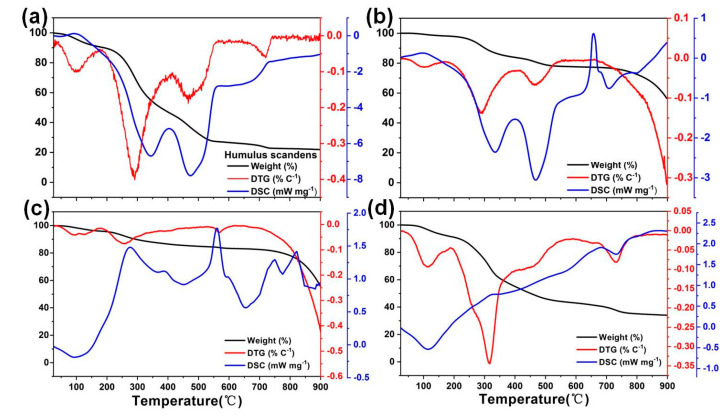
TG, DTG and DSC pyrolysis curves for (**a**) pure HS, (**b**) HS and salt and (**c**) HS with salt and NaOH in the air; (**d**) the pyrolysis of HS under N_2_ protection.

The pyrolysis process of HS biomass in the air or in a nitrogen atmosphere, and the pyrolysis process of HS in mixed salt with and without NaOH in the air were studied by using a synchronous thermal analyzer. Figure 1 shows the corresponding TG, DSC and DTG curves. For the pyrolysis process of HS in the air, Figure 1a shows that the first ~10% of weight loss ends around ~170 °C, which corresponds to the evaporation of moisture and decomposition of organic matter in HS. The second weight loss stage occurs in the range of 170–420 °C, with a total weight loss of ~55% due to the pyrolysis of organic compounds and the burning of carbonized products. When the temperature rose to ~600 °C, the weight loss of HS reached the maximum of ~76.8%. When the temperature was elevated to 730 °C, the weight of HS did not change, which indicates that the organic components in HS are completely removed at high temperature without a salt seal. The residue corresponds to ~23.2% of the initial biomass. Based on Figure 1b, the weight loss under N_2_ is controlled at ~67.6%, indicating that ~32.4% of the biomass was retained after the calcination. Compared with that of the sample test in the air, it could be concluded that many mineral species are present in the biochars. For the mixture of HS and salt at a mass ratio of 1:2, it is found in Figure 1c that the weight loss of HS in the first three stages is basically the same as that in Figure 1a. Based on the calculation, the biomass weight loss at 600 °C is at ~60%, which is much lower than that without the salt protection (~75.0%), indicating the salt sealing partially reduces the hydrolysis of the biomass. As depicted in Figure 1d, after the addition of NaOH, it can be observed that the weight loss experiences a slight increase from ~60% to ~64%, which indicates that the NaOH helps to cave the carbon structure during the high-temperature reaction. Based on these observations, a possible formation mechanism is proposed: due to the etching effect of the molten salt ions as well as the penetrated O_2_ atoms at high temperature, marco- and meso-porous structures form in the biochar. In addition, the NaOH not only further increases the pore diameters, but also introduces abundant oxygen functional groups on the biochar surface.

**Figure 2:**
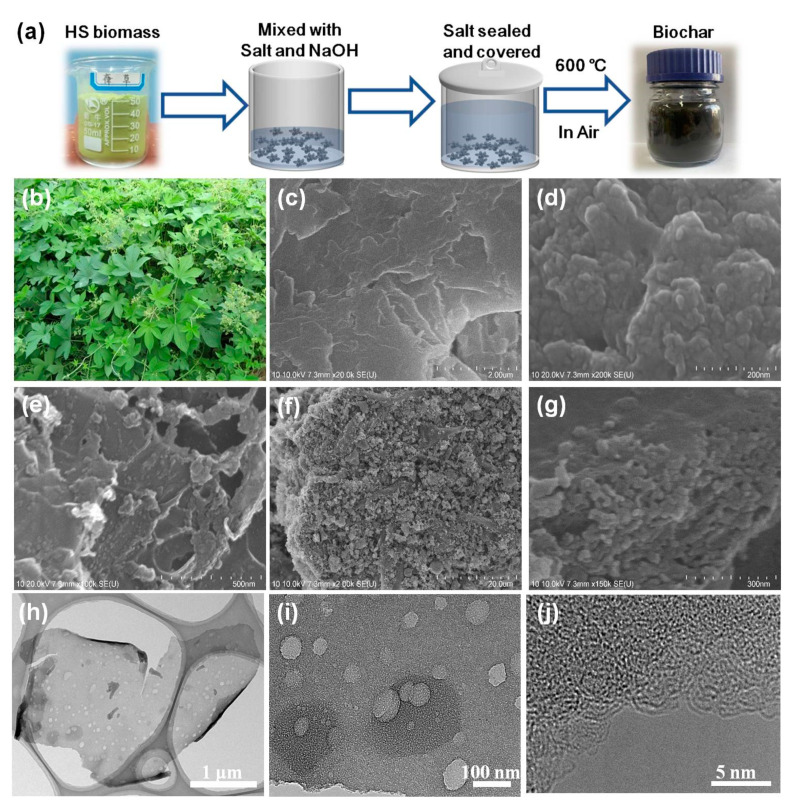
(**a**) Illustration of the preparation process of the biochar, (**b**) HS plant growth in the north of China, the inset is the corresponding ground powders. FE-SEM image of (**c**) the pristine HS biomass, (**d**) HSC-N_2_, (**e**) HSC-M, and (**f**,**g**) HSC-MA-2 with different magnifications. (**h**,**i**) TEM images with different magnifications and the (**j**) HRTEM image.

Figure 2a shows an illustration of the preparation process of the HS-derived biochars. After the biomass was ground into a green powder, it was mixed with NaOH and the KCl/NaCl salt mix, and then sealed with KCl/NaCl in a crucible. Afterwards, the crucible was covered and heated to a given temperature for the pyrolysis. Finally, the product was washed with water to remove the excessive NaOH and salt. The biochars was dried for the adsorption of the heavy metal ions. The morphology and microstructure of the HS-derived biochars were studied using a field emission scanning electron microscope (FE-SEM). Figure 2b illustrates the excessive growth of HS in the north of China, which causes negative impacts on both agricultural production as well as the human activities. Figure 2c shows the FE-SEM image of the original biomass, indicating that the surface is smooth, with almost no pore structure detected. As depicted in Figure 2d, the surface of the biochar prepared in a nitrogen atmosphere is rougher, demonstrating that carbonation resulted in the formation of a porous structure in the as-yielded biochars (Supplementary Materials Figure S3). However, the porosities are closely packed together. However, as seen in Figure 2e, under the protection of molten salt, the HSC-MS exhibits an irregular sheet-like structure with a rougher surface. As shown in Supplementary Materials Figure S4, after the dual activation of molten salt and NaOH, the surfaces of the HSC-MAs all become rougher. In addition, the overall particle size of the HSC-MA-2 becomes smaller and the surface shows greater roughness, forming a rich porous structure (Figure 2f,g). Such porous structures allow the biochar to have large specific surface area, which can facilitate the mass diffusion in the pore channels of the biochar. The above results show that the formation of the porous structure of biomass results from the etching effects of both the molten salt and NaOH at high temperature. Meanwhile, the abundant porous structure and large specific surface area can promote the adsorption of heavy metal pollutants on the HS-derived biochars. Figure 2h depicts the TEM image of HSC-MA-2. The sample is composed of thin sheets with a large number of macropores with diameters of 50–100 nm. The TEM image with higher magnification (Figure 2i) shows a great number of mesoporous and micropores, due to the NaOH-assisted pyrolysis. The high-resolution TEM (HRTEM) image shown in Figure 2j indicates that many wrinkling lattice fringes can be detected at the surface of the carbon, indicating that the surface of the biochar was highly graphitized.

**Figure 3:**
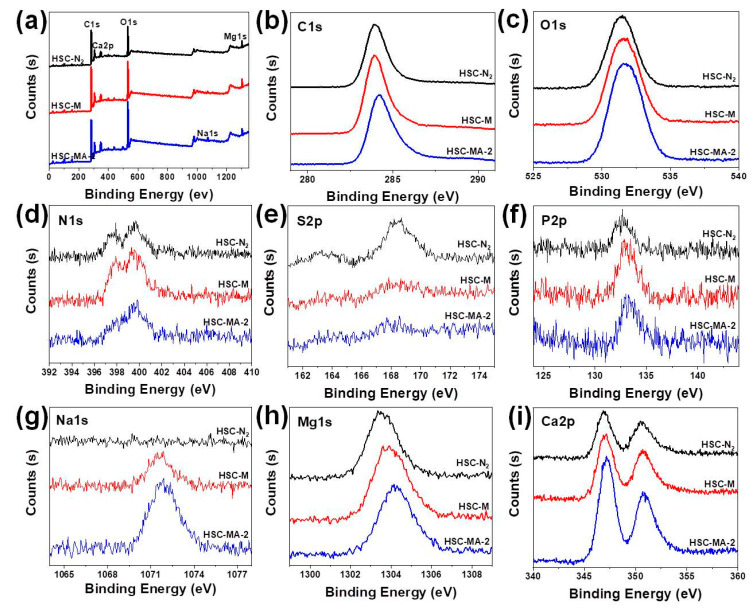
XPS image of the HSC-N_2_, HSC-M and HSC-MA-2 (**a**) full spectra, (**b**) C1s, (**c**) O1s, (**d**) N2p, (**e**) S2p, (**f**) P2p, (**g**) Na1s, (**h**) Mg1s, (**i**) Ca2p.

X-ray photoelectron spectroscopy (XPS) was used to analyze the chemical elements of the biochars. Figure 3a shows the survey analysis of the HSC-N_2_, HSC-M and HSC-MA-2. There are two distinct peaks corresponding to C1s and O1s at the binding energies of 284.6 and 532.4 eV in the spectrum. In addition, HSC-MA-2 shows a much enhanced content of mineral species such as Na, Mg and Ca. The detailed element compositions are summarized in Table 1.

The C1s peak shown in Figure 3b indicates that HSC-N_2_ and HSC-M have a similar relative carbon content, while HSC-MA-2 has a relatively low carbon content, suggesting that the etching of the molten salt and NaOH could substantially reduce the carbon sp^2^ domain and introduce abundant oxygen motifs. Based on the fitting of the C1s signals shown in Supplementary Materials Figure S5, it can be also observed that the peaks belonging to C-C and C=C (at 284.8 eV and 286.5 eV) are reduced. Meanwhile, the peaks ascribed to C=O and COOH (at 288.7 eV and 290.9 eV) are slightly enhanced, and the increased binding energy suggests that carbon motifs with high valence forms are incorporated in the HSC-MA-2 [32,33].

Moreover, this change can be also detected in the O1s spectrum displayed in Figure 3c. It is obvious that the content of oxygen in HSC-MA-2 is the highest, which indicates that the oxygen functional groups are introduced into the biochar by the NaOH modification reagent. The deconvolution of O 1s spectra shown in Supplementary Materials Figure S6 can be fit into four types: C=O (531.3 eV), OH/RCOOR (532.6 eV), O-C=O (533.7 eV) and COOH (534.9 eV). Compared with that of HSC-N_2_ and HSC-M, the peak ascribing to COOH is greatly enhanced in HSC-MA-2, indicating that carboxyl groups are successfully introduced into the biochars. This result is in good agreement with the C1s spectrum, and the increased carboxyl group population could in turn enhance the surface coordination effects on the heavy metal ions.

Figure 3d–f shows the contents of N, S and P in biochar. It can be seen that the biochars prepared from HS all contain a certain amount of N, P and S. Considering that no N-, P- and S-containing regents were used in the preparation, these heteroatoms originate from the pristine tissue of the HS biomass. Based on the previous reports, N, P and S with a lone pair of electrons show high affinity with the positively charged heavy metal ions [34]. Although HSC-MA-2 experience rigid MS and NaOH etching, the N and P could still be preserved in the biochar, which could further enhance the surface affinity towards the Pb and Cu ions. As depicted in Figure 3g–i, the modification of NaOH can greatly increase the content of Na, Mg and Ca in the biochar (the detail composition values are summarized in Table 1). These mineral ions are beneficial to the adsorption of heavy metals via the ion exchange process. For these biochars, no signal belonging to Pb and Cu could be detected, as shown in Supplementary Materials Figures S7 and S8.

In order to better study the element content in the biochars, we used inductively coupled plasma mass spectrometry (ICP-MS) to better reveal the metallic elements of the biochars. The detail results are shown in Table 2, and it is clear that the as-measured composition is much higher compared with that measured using XPS. Considering that XPS mainly detects the elements distributed on the surface of the biochars, the different composition results of ICP-MS demonstrate that the majority of the mineral species are embedded inside the biochars. Furthermore, HSC-MA-2 exhibits the highest contents in Na, Mg and Ca, which is in accordance with the XPS measurement.

**Figure 4:**
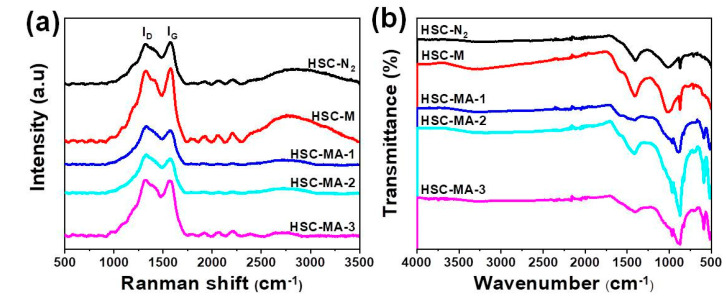
Raman (**a**) and FTIR (**b**) spectra of HSC-N_2_, HSC-M, HSC-MA-1, HSC-MA-2, and HSC-MA-3.

In order to analyze the graphitization degree and crystal phase of the biochars, the biochars were analyzed using laser Raman spectra, as shown in Figure 4a. There are characteristic diffraction peaks at ~1320 and ~1570 cm^−1^, corresponding to the D band (disordered band) and G band (graphitized band) of the biochars [11]. The D-band is due to the defects and disorder induced by carbon atoms hybridizing, and the G-band is due to the stretching of carbon atoms. In general, the intensity ratio (I_D_/I_G_) of the D and G bands is used to describe the disorder degree of biochars. The calculated D and G band intensity ratios of HSC-N_2_, HSC-M, HSC-MA-1, HSC-MA-2, HSC-MA-3 are 0.98, 0.99, 1.02, 1.04 and 1.01, respectively. The order of I_D_/I_G_ was HSC-N_2_ < HSC-M < HSC-MA-3 < HSC-MA-1 < HSC-MA-2, which indicated that the stable sp^2^ domain of the carbon skeleton is etched and many surface defects are formed in HSC-MA-2. The increased disorder degree could permit the enhanced adsorption performance of the material due to the high surface energy.

Fourier transform infrared spectrometry (FTIR) is an important technique to identify the functional groups on the surface of adsorbents. As shown in Figure 4b, characteristic peaks belonging to C-O motifs are detected (C-O, 956 cm^−1^ (stretching tensile vibration), 1034 cm^−1^ (OH, hydroxyl stretching vibration), 1124 cm^−1^ (C-O-C, stretching vibration), 1301 cm^−1^ (COOH or CHO bending vibration) and 1560 cm^−1^ (C=O, stretching vibration)). Therefore, the three peaks centered at ~950, ~1000 and ~1300 cm^−1^ could be attributed to the C-O, OH and COOH or CHO groups, respectively. It can be seen that the intensity of oxygen functional group peaks, such as COOH or CHO, are greatly increased in HSC-MA-2 compared with other samples. The increased oxygen functional groups could provide abundant coordinating sites for the heavy metal ions on the biochars, which could greatly facilitate the surface adsorption process. [12,35]

#### Surface Area and Pore Structure

**Figure 5:**
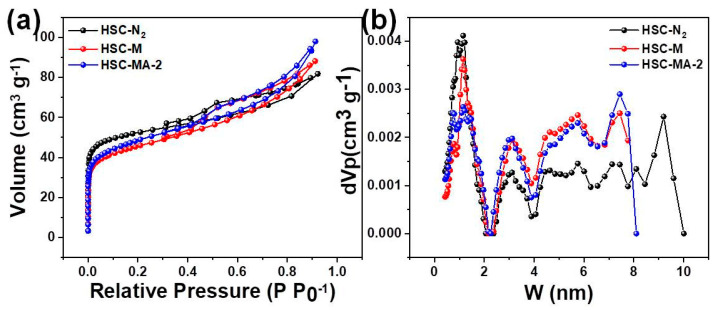
(**a**) Nitrogen adsorption/desorption analysis and the (**b**) pore size distribution of the HSC-N_2_, HSC-M and HSC-MA-2.

The pore structures of HSC-N_2_, HSC-M and HSC-MA-2 were studied by means of the nitrogen adsorption/desorption method. Figure 5 shows the nitrogen adsorption/desorption isotherms of the biochars, which belongs to the mixture of the type I and type IV isotherms, indicating the co-existence of the micro- and meso-porous structures. The micropore adsorption initially occurs at a low pressure. The amount of nitrogen adsorbed is between 0.4 and 0.9, corresponding to the capillary condensation of meso-pores, which indicates that HSC-MA-2 has a hierarchical porous structure [36]. In the range of relative pressure greater than 0.9, the nitrogen desorption isotherm of the HSC-MA-2 shows a continuous rising trend compared with that of the other two curves, indicating that there are still large pores in the biochar. The results of nitrogen adsorption and desorption show that a lot of micro- and meso-pores with different size distributions can be detected in the biochars prepared by molten salt with NaOH. As listed in Table 3, the BET specific surface area of the biochar experiences a slight reduction from 182.9 to 161.4 and 171.9 m^2^/g. However, the overall pore volume and pore diameter increase with the addition of NaOH. The results indicate that the optimal HSC-MA-2 exhibits well-developed pore structures with a greater pore diameter and larger pore volume, which could benefit the mass diffusion and facilitate the adsorption process on the exposed inner surfaces.

### 3.2. Adsorption Tests

**Figure 6:**
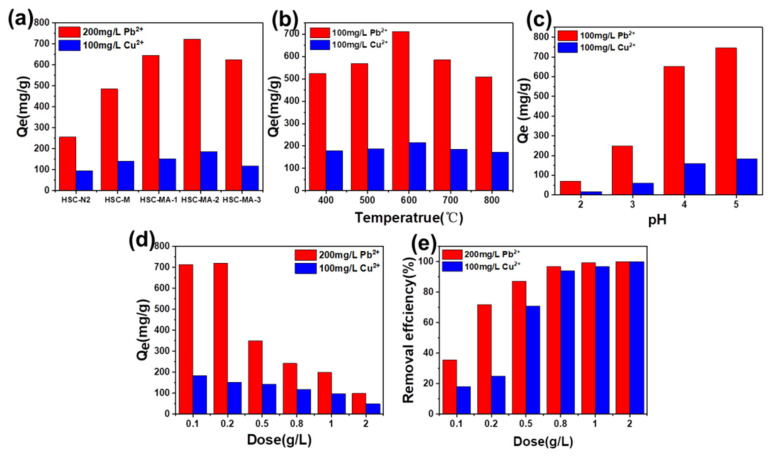
Influence of the modifying agents (**a**), temperatures (**b**), pH (**c**) and the dosage (**d**,**e**) on the adsorption capacity and removal efficiency of Pb^2+^ and Cu^2+^.

The adsorption of Pb^2+^ and Cu^2+^ on different biochars were studied. In general, the Pb^2+^ adsorption capacity of biochar prepared with HS (Figure 6a) is much higher than that of Cu^2+^, which indicates that the as-prepared biochar has better affinity to Pb^2+^. In addition, the adsorption capacity increases with the NaOH amount, and the maximum value was reached when the mass ratio of biomass to NaOH equaled 3/2. As shown in Figure 6b, the pyrolysis temperature was controlled at 400, 500, 600, 700 and 800 °C to study the temperature influences. Obviously, the biochar prepared at 600 °C demonstrated high adsorption capacity, indicating that 600 °C is the optimal pyrolysis temperature for HS biomass.

It is generally believed that the pH value affects the states of metal ions, the ionization degree of the solution as well as the surface charge of biochars. It is of great significance to study the effects of pH on adsorption performances [37]. To avoid precipitation, the pH’s effects on the Pb^2+^ and Cu^2+^ ions adsorption capacity of the biochars were investigated at the pH range of 2.0–5.0 (Figure 6c). The adsorption capacity of the Cu^2+^ and Pb^2+^ increased with increasing pH values. At a low pH, the competition adsorption of H^+^ on the surface sites greatly limit the adsorption capacity of Pb^2+^ and Cu^2+^ of the biochar. To make it worse, some minerals in the biochar may also dissolve and release large amounts of cations, such as Ca^2+^ and Mg^2+^. These released ions may compete with Pb^2+^ and Cu^2+^ for the adsorption sites, hindering the adsorption capacity of heavy metal ions. Thus, the sorption kinetics and sorption isotherm experiments were carried out at a pH of 5.

The effects of HSC-MA-2 with different dosages on the adsorption capacities (Figure 6d) and removal rates (Figure 6e) were studied to determine the optimal dosage. As expected, the adsorption capacity decreased with the increase in the biochar dosage. For Pb^2+^, the removal efficiency was ~75% when the dosage was 0.2 g/L, and reached above ~90% when the dosage was elevated to 0.5 g/L. For Cu^2+^, the removal efficiency reached over ~75% when the dosage was 0.5 g/L, and ~90% removal efficiency was achieved when the dosage was 1.0 g/L. Based on these analyses, the best dosages of Pb^2+^ and Cu^2+^ adsorbed by HSC-MA-2 are 0.2 g/L and 0.5 g/L, respectively. Considering that HSC-MA-2 has the best adsorption effect on Pb^2+^ and Cu^2+^, the subsequent adsorption mechanism studies will focus on HSC-MA-2, adopting HSC-N_2_ and HSC-M as references.

#### 3.2.1. Adsorption Kinetics

**Figure 7:**
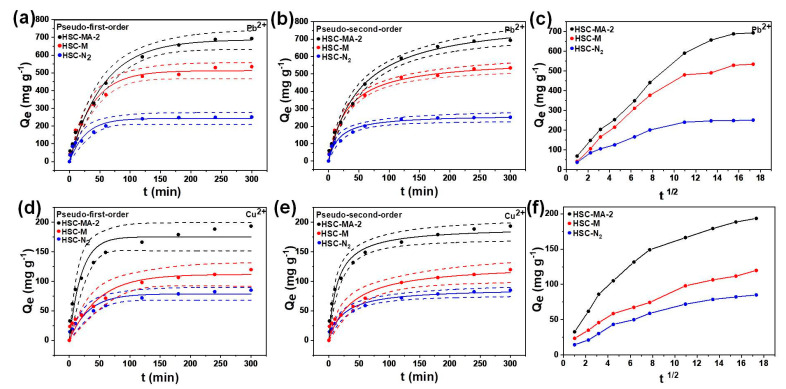
(**a**,**b**) Adsorption kinetics of Pb^2+^ on HSC-MA-2, HSC-M and HSC-N_2_ (linear plot of pseudo-first-order and pseudo-second-order rate equation); (**d**,**e**) adsorption kinetics of Cu^2+^ on HSC-MA-2, HSC-M and HSC-N_2_; intra-particle diffusion model for (**c**) Pb^2+^ and (**f**) Cu^2+^.

The adsorption kinetics of Pb^2+^ and Cu^2+^ adsorption on different biochars are shown in Figure 7a–e. Table 4 depicts the kinetic parameters of the adsorption of Pb^2+^ and Cu^2+^. The correlation coefficient (R^2^) derived from the PSO model is much higher than that of the PFO model, which indicates that the adsorption of heavy metal ions is mainly dominated by the chemical adsorption, such as complexation and precipitation [4,5]. The adsorption rate is very fast at the beginning of the adsorption process, and then gradually reduces with the increase in contact time, until the adsorption equilibrium is reached after ~120 min.

The fitted curves of the intra-particle diffusion model are shown in Figure 7c,f. The intra-particle diffusion analysis shows that the adsorption of Pb^2+^ and Cu^2+^ by HSC-MA-2, HSC-M and HSC-N_2_ can be divided into three stages. Rapid adsorption rates are observed at the first stage, which results from the presence of many adsorption sites on the biochar surface [24,37,38,39]. At the second stage, the adsorption is controlled by the intra-particle diffusion rate. Due to the decrease in heavy metal ion concentration and the very low mass transfer rate, the intra-particle diffusion process is slower than the first stage. The third stage is the equilibrium stage, and the adsorption rate is greatly reduced [38]. Based on these observations, the HSC-MA-2 shows the fastest adsorption rate among the samples, indicating that the well-developed porous structure could benefit the intra-particle diffusion, thus enhancing the adsorption kinetics.

#### 3.2.2. Adsorption Isotherm

**Figure 8:**
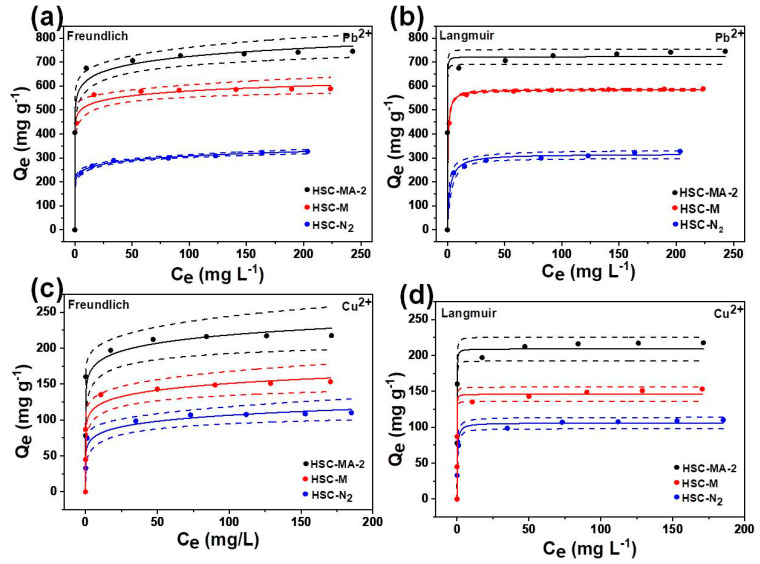
Freundlich sorption isotherms of (**a**) Pb^2+^ and (**c**) Cu^2+^; and Langmuir sorption isotherms of (**b**) Pb^2+^ and (**d**) Cu^2+^ on the HSC-N_2_, HSC-M and HSC-MA-2.

The effects of the initial concentration of Pb^2+^ and Cu^2+^ on adsorption capacity were investigated. Due to the concentration gradient between the solution and the adsorbent surface, the adsorption capacity increases with the initial concentration of Pb^2+^ and Cu^2+^. It is generally believed that a higher initial concentration of Pb^2+^ and Cu^2+^ provides a greater driving force to overcome the mass transfer resistance in the solution, which results in a higher adsorption capacity. When the initial concentrations of Pb^2+^ and Cu^2+^ are higher, the adsorption capacity is prone to reach a balance due to the limitation of the maximum adsorption capacities [39]. Freundlich and Langmuir models were used to describe the relationship between adsorption capacity and equilibrium concentration, and the fitting curves are shown in Figure 8. The as-calculated Q_max_, correlation coefficient (R^2^), and other relevant constants are displayed in Table 5. The Langmuir isotherm has higher R^2^ than the Freundlich model, which further proves that the chemical adsorption governs the removal process. The Q_max_ of HSC-MA-2 is 748.1 and 221.1 mg/g for Pb^2+^ and Cu^2+^, respectively. These results are in good agreement with the above experimental results, indicating that the adsorption capacity of the modified biochar for Pb^2+^ and Cu^2+^ is significantly improved compared to the biochar prepared directly by the molten salt sealing method without the modification reagent (594.5 and 155.0 mg/g for Pb^2+^ and Cu^2+^) or under N_2_ protection (310.6 and 110.0 mg/g for Pb^2+^ and Cu^2+^). These results further indicate that under the conditions of an air atmosphere and high temperature, the etching effects of molten salt and NaOH on carbon products can endow the biochar with high specific surface area and rich oxygen functional groups, which is conducive to the adsorption capacity of heavy metal ions. In addition, as shown in Table 6, the optimized HSC-MA-2 demonstrates much enhanced adsorption capacity compared with that of the previously reported biochars prepared from different biomasses [40,41,42,43,44,45,46,47,48].

#### 3.2.3. Effects of Competition Ions

**Figure 9:**
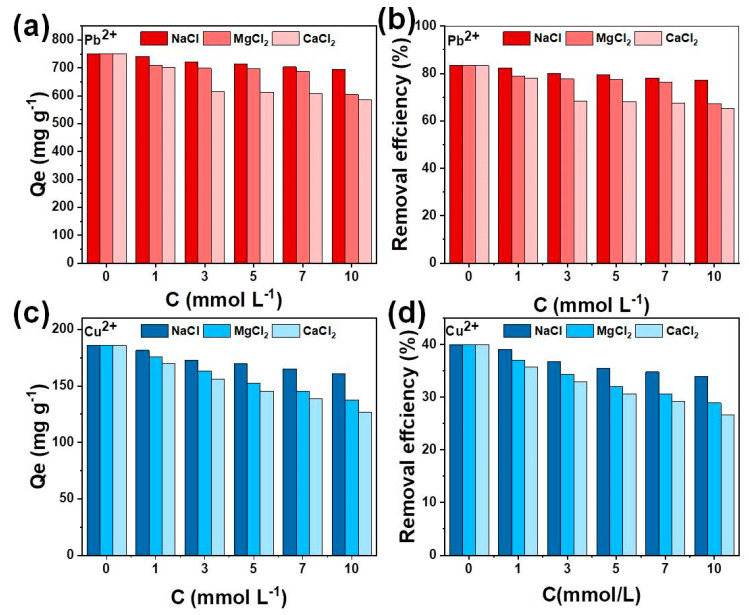
The effect of background ions on the sorption of (**a**) HSC-MA-2 and (**b**) HSC-N2 on Pb^2+^, and (**c**) HSC-MA-2 and (**d**) HSC-N2 on Cu^2+^.

In the adsorption process, the background ions (Na^+^, Mg^2+^ and Ca^2+^) affect the adsorption of target ions on the biochar. As shown in Figure 9, the results indicate that Na^+^ has little effect on the removal of Pb^2+^ and Cu^2+^, while the presence of Mg^2+^ and Ca^2+^ has a greater influence on the adsorption capacity of HSC-MA-2 compared with Na^+^ (monovalent cation). As the concentration of background ions is increased, the removal efficiencies of Pb^2+^ and Cu^2+^ decrease due to the enhanced ion strength of Mg^2+^ and Ca^2+^, which could be more competitive for the active sites on HSC-MA-2. In addition, the influence of Ca^2+^ on the adsorption of Pb^2+^ and Cu^2+^ is greater than that of Mg^2+^. The higher valence sates of Mg^2+^ and Ca^2+^ may lead to stronger hydration, which makes Mg-OH more stable than Ca^2+^ in aqueous solutions and thus reduces its interaction with the surface active sites [39]. Therefore, the effect of Mg^2+^ on Pb^2+^ and Cu^2+^ adsorption by HSC-MA-2 is lower than that of Ca^2+^, but greater than that of Na^+^.

#### 3.2.4. Multi-Metal Adsorption and Column Test

**Figure 10:**
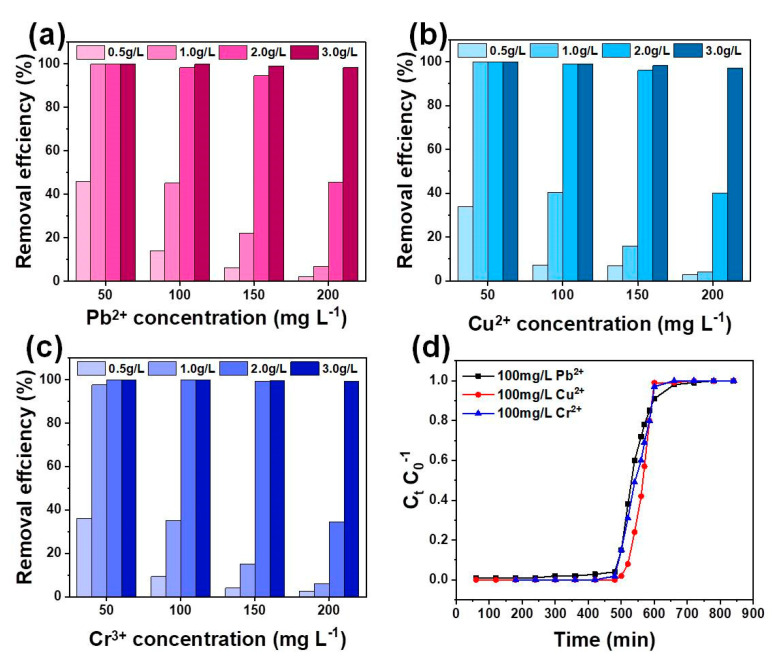
The effects of the absorbent dosages and the initial concentration of the solution coexisting with (**a**) Pb^2+^, (**b**) Cu^2+^ and (**c**) Cr^3+^ on the removal efficiency of each metal ion, (**d**) kinetics of column adsorption of heavy metal ions in practical wastewater.

The interaction of coexisting heavy metal ions in the adsorption process has more practical significance than in the single heavy metal ion system. Therefore, the adsorption capacity in the poly-metal ion system was studied (Figure 10a–c). As expected, the removal efficiencies of Pb^2+^, Cu^2+^, and Cr^3+^ increase with the increase in the biochar dosage. For HSC-MA-2, when the biochar dosage is increased from 50 to 300 mg, the removal efficiency of all heavy metal ions is enhanced significantly. The removal efficiency of all heavy metal ions decreases with the increase in the concentration of metal ions. As the dosage of HSC-MA-2 is increased to 300 mg, the removal efficiency of all heavy metal ions reaches ~100%. These results suggest that when the metal ions compete for the same adsorption sites, the metal ions with higher affinity replace the other ions with lower affinity, and the most intense competition takes place when there is a high concentration of heavy metal ions. For HSC-MA-2 (200 mg), the removal efficiencies of Pb^2+^, Cu^2+^ and Cr^3+^ are 98.05%, 94.57% and 96.18%, respectively, when the concentration of all metal ions is set at 150 mg/L. It is evidenced that the presence of Cr^3+^ has a significant effect on the adsorption of Pb^2+^ and Cu^2+^ on the biochar, due to the strong affinity for Cr^3+^. The experimental results prove that the HSC-MA-2 can effectively take up many types of heavy metal ions at the same time. Based on the abovementioned batch experiments, column adsorption experiments are also carried out in order to investigate the possible application of HSC-MA-2 in large-scale operations for the practical wastewater treatment. The column adsorption experiments were conducted using HSC-MA-2 as the filler (Figure 10d). The simulated wastewater was prepared from the Yellow River; 100 mg/L Cu^2+^, Pb^2+^ and Cr^3+^ were added, and the average discharge of the column was adjusted to 0.4 mL/min. As the operation time increases, heavy metal ions adsorption gradually decreases. This phenomenon indicates that HSC-MA-2 is gradually saturated regarding heavy metal ions’ adsorption capacity. Cu^2+^ and Cr^3+^ become saturated within 600 min, while the transition of Pb^2+^ occurs at 500 min and reaches saturation at 720 min. Generally, the longer the saturation time, the better the adsorption effect of the metal ions. Therefore, in the simulated column adsorption of practical wastewater, the affinity of HSC-MA-2 to different heavy metal ions follows the sequence of Pb^2+^ > Cu^2+^ > Cr^3+^. Considering the outstanding removal effects for the heavy metal ions, the HSC-MA-2 provides a highly efficient removal rate for the practical wastewater treatment.

### 3.3. Adsorption Mechanism

**Figure 11:**
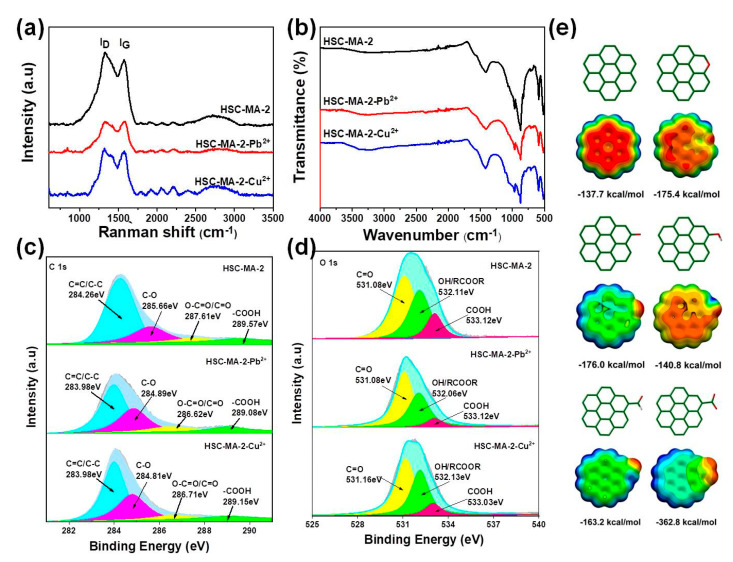
Raman (**a**), FTIR (**b**) spectra, high-resolution C1s (**c**) and O1s (**d**) spectra of HSC-MA-2 before and after Pb^2+^ and Cu^2+^ adsorption, (**e**) Gauss simulation on the Pb^2+^ binding energies with different types of oxygen functional groups on the carbon domain.

As indicated in previous reports, the sorption mechanism probably involved surface complexation, metal precipitation, ion exchange, π–π interactions, and physical sorption [39,42]. In order to investigate the high sorption capacity of HSC-MA-2, the biochars before and after metal ion adsorption were investigated in detail. As shown in Figure 11a, the Raman signal is greatly subtracted after the adsorption of the heavy metal ions, especially for the case of the Pb^2+^ adsorption, which indicates that the signal is greatly affected by the interaction between the biochar and the positively charged heavy metal ions. The reduced I_G_ signal reflects the deviation of delocalized π electrons in the carbon domain, suggesting the strong π–π interaction between the biochar and the metal ions. In particular, the I_D_ intensity is much more reduced than that of the I_G_, which indicates that the defects remaining in the carbon domain serve as the most effective sites for the adsorption. This phenomenon can be further proved by the FTIR spectra. As depicted in Figure 11b, the absorption peaks centered at ~950, ~1000 and ~1300 cm^−1^ are greatly abated, which is also attributed to the coordination of the oxygen group with the metal ions. Furthermore, the high-resolution XPS spectra on the C1s and O1s are disclosed in Figure 11c,d. The reduced C1s peaks can be explained by the strong interaction on the surface, which results from the heavy adsorption loading of the metal ions on the biochar. In addition, the O1s can be delineated into three peaks, corresponding to the OH, C=O and COOH, respectively. It is obvious that after the Pb^2+^ adsorption, the peaks ascribed to C=O and COOH are greatly reduced, which indicates that the C=O and COOH groups play significance roles in the heavy metal adsorption. Based on the Gauss simulation, COOH and C=O more effectively lead to the charge polarization on the carbon surface, which gives rise to the higher surface affinity towards the metal ions (362.8 kcal/mol) (Figure 11e) compared with the carbon domain (−137.6 eV) and other oxygen functional groups (C-O-C (−175.4 eV), C=O (−176.0 eV), OH (−140.8 eV) and H-C=O (−163.2 eV)). The computation results indicate that the COOH and C=O motifs demonstrate the most enhanced binding energy towards the Pb^2+^, which agrees well with our experimental findings.

**Figure 12:**
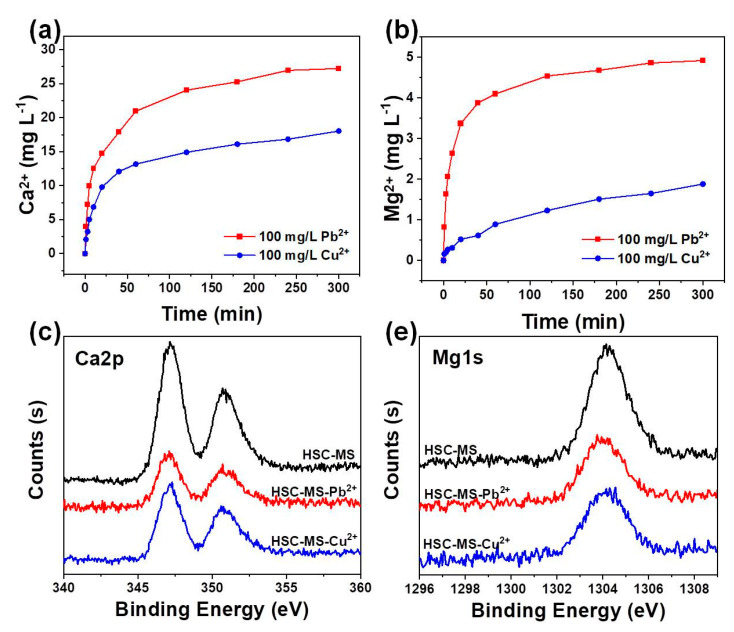
The concentration of Ca^2+^ (**a**) and Mg^2+^ (**b**) released in the adsorption process. High-resolution XPS spectra on the (**c**) Ca2p and (**d**) Mg1s of HSC-MA-2 before and after Pb^2+^ and Cu^2+^ adsorption.

In order to investigate the ion exchange during the adsorption process, the release of Ca^2+^ and Mg^2+^ ions was monitored during the adsorption process (Figure 12a,b). The Ca^2+^ and Mg^2+^ concentrations experience continuous enhancement with prolonged adsorption times, which suggests that the release of Ca^2+^ and Mg^2+^ is accompanied by the heavy metal ion adsorption. In addition, the amount of Ca^2+^ released is much higher than that of the Mg^2+^, implying that Ca^2+^ is the most effective mineral species helping the adsorption process. Moreover, the high-resolution XPS spectra shown in Figure 12c,d indicate that after the Pb^2+^ and Cu^2+^ adsorption, the Ca^2+^ and Mg^2+^ content in the spectra are greatly suppressed, especially for the Pb^2+^ adsorption case. Taking into account the reduced Ca^2+^ and Mg^2+^ remaining in the biochar, as well as the enhanced concentration of Ca^2+^ and Mg^2+^ ions in the final adsorption system, it can be concluded that ion exchange plays an important role in facilitating heavy metal ion adsorption.

## 4. Conclusions

The NaOH-modified molten salt method was adopted to prepare a series of biochars using *Humulus scandens* as a biomass precursor. The optimized biochar HSC-MA-2 exhibits excellent Pb^2+^ and Cu^2+^ adsorption performance, and the Q_m_ can reach 748 and 221 mg/g, respectively. In addition, HSC-MA-2 shows good anti-interference ability and high removal efficiency in the simulated wastewater using Yellow River water for practical application. The enhanced performance of the HSC-MA-2 can be ascribed to the following qualities of the proposed NaOH-modified molten salt process. Firstly, the synthetic etching effect of the molten salt and the NaOH media introduce a large surface area and a large porous structure to offer abundant adsorption sites as well as mass diffusion channels. Secondly, the penetrating O_2_ molecules and the NaOH endow the carbon surface with a large population of the oxygen functional groups such as C=O and COOH, which substantially increase the coordinating strength with the positively charged metal ions. Finally, the NaOH helps to preserve the pristine mineral species from the biomass in the biochar, which serve as the active centers to adsorb the foreign heavy metal ions through ion exchange. Therefore, this method could serve as a general protocol to produce high-performance biochar for water treatment.

## Figures and Tables

**Table 1:** The element composition of the biochars measured by XPS.

Element	C (%)	O (%)	N (%)	P (%)	S (%)	Na (%)	Mg(%)	Ca (%)
HSC-N_2_	60.23	24.53	3.69	0.79	1.28	0	2.82	3.28
HSC-M	60.67	24.45	3.65	1.12	0	0.71	2.93	3.36
HSC-MA-2	54.48	28.75	2.02	1.38	0	1.63	5.25	5.22

**Table 2:** The element composition of the biochar measured by ICP-MS.

Element (mg/g)	Na_23_	Mg_24_	Ca_43_
HS	0.89	8.45	9.26
HSC-N_2_	3.48	22.60	17.00
HSC-M	3.19	26.40	23.70
HSC-MA-2	3.33	41.50	30.10

**Table 3:** BET surface specific area (SSA) and pore structure of biochars.

Sample	SSA (m^2^/g)	Mean Pore Diameter (nm)	Total Pore Volume (cm^3^/g)
HSC-N_2_	182.9	2.77	0.13
HSC-M	161.4	3.38	0.14
HSC-MA-2	171.9	3.52	0.15

**Table 4:** Kinetic parameters of Pb^2+^ and Cu^2+^ on HSC-N_2_, HSC-M and HSC-MA-2.

Sample		Pseudo-First-Order			Pseudo-Second-Order		
		R^2^	Q_e_	K_1_	R^2^	Q_e_	K_2_
Pb^2+^	HSC-MS	0.9870	512.5411	0.2627	0.9943	578.1483	0.0100
	HSC-MS-2	0.9940	687.8283	0.2798	0.9946	729.8656	0.0100
	HSC-N_2_	0.9625	242.9751	0.1138	0.9757	266.1626	0.0100
Cu^2+^	HSC-MS	0.9525	111.7617	0.0208	0.9822	129.4631	0.0100
	HSC-MS-2	0.9609	161.0000	0.0655	0.9664	192.0009	0.0100
	HSC-N_2_	0.9636	78.6980	0.0319	0.9819	88.1899	0.0100

**Table 5:** Langmuir and Freundlich constants and correlation coefficients (R^2^) for Pb^2+^ and Cu^2+^ adsorption on HSC-N_2_, HSC-M and HSC-MA-2.

Sample		Langmuir			Freundlich		
		R^2^	Q_m_	K_L_	R^2^	N_f_	K_f_
Pb^2+^	HSC-MS	0.9999	594.4691	1.0000	0.9916	20.2338	460.9877
	HSC-MS-2	0.9932	748.0998	1.0000	0.9900	13.0424	502.6869
	HSC-N_2_	0.9913	310.6050	0.4741	0.9984	12.0606	210.2611
Cu^2+^	HSC-MS	0.9814	155.0275	1.0000	0.9622	9.8353	96.2226
	HSC-MS-2	0.9879	221.0786	1.0000	0.9696	12.7665	152.2614
	HSC-N_2_	0.9856	110.0282	1.0000	0.9656	8.4288	61.7383

**Table 6:** The comparison on the adsorption performances.

Biomass	Target Ions	Q_max_	SSA	References
soybean cake	Pb^2+^	133.60 mg/g	32.7 m^2^/g	[22]
Shell	Pb^2+^	100.25 mg/g	499.2 m^2^/g	[24]
Medulla tetrapanacis	Cu^2+^, Pb^2+^	Cu^2+^ 458.72 mg/g Pb^2+^ 1031.23 mg/g	246.85 m^2^/g	[39]
Enteromorpha	Cu^2+^, Pb^2+^	Cu^2+^ 254 mg/g Pb^2+^ 98 mg/g	29.7 m^2^/g	[40]
corn stalks	Cu^2+^	152.61 mg/g	4.46 m^2^/g	[41]
Rice husks	Cu^2+^	265 mg/g	2330 m^2^/g	[42]
Palm	Pb^2+^	118.08 mg/g	Not given	[21]
sugar cane	Pb^2+^	86.96 mg/g	92.30 m^2^/g	[43]
water hyacinths	Pb^2+^	128.95 mg/g	51.15 m^2^/g	[44]
Enteromorpha compressa	Cu^2+^	137 mg/g	52 m^2^/g	[15]
sesame straw	Cu^2+^, Pb^2+^	Cu^2+^ 55 mg/kg Pb^2+^ 102 mg/kg	Not given	[45]
fresh banana peels	Cu^2+^, Pb^2+^	Cu^2+^ 24.27 mg/g Pb^2+^ 193 mg/g	82.4 m^2^/g	[46,47]
corn stalk	Cu^2^, Pb^2+^	Cu^2+^ 161.9 mg/g Pb^2+^ 195.1 mg/g	603.4 m^2^/g	[48]

## Data Availability

The detailed data in the study are available from the corresponding authors by request. (Ning Liu, Dapeng Wu and KaiJiang).

## Supplementary Materials

The following are available online at https://www.mdpi.com/article/10.3390/nano11123255/s1, Figure S1 Location of the site where the HS biomass is obtained. (The map is downloaded from an open source website: https://www.google.com/maps/); Figure S2 Location of the site where the Yellow river water is fetched. (The map is downloaded from an open source website: https://www.google.com/maps/); Figure S3 SEM images of HS, HSC-N2, HSC-M; Figure S4 SEM images of HSC-MA-1, HSC-MA-2, HSC-MA-3; Figure S5 detailed XPS analysis on the C1s of the biochars; Figure S6 detailed XPS analysis on the O1s of the biochars; Figure S7 XPS on the Cu2p of the biochar before adsorption; Figure S8 XPS on the Pb4f of the biochar before adsorption.
