# Supplementary material for: Humulus scandens-Derived Biochars for the Effective Removal of Heavy Metal Ions: Isotherm/Kinetic Study, Column Adsorption and Mechanism Investigation
Source: Nanomaterials (Basel). 2021 Nov 30;11(12):3255. doi: 10.3390/nano11123255 (PMC8704399; doi:10.3390/nano11123255)
Supplement: Supplementary file 1 [file nanomaterials-11-03255-s001.zip › nanomaterials-1444568-supplementary.pdf]

Supplementary Materials

Humulus Scandens Derived Biochars for Effective Removal of Heavy Metal Ions: Isotherm/kinetic Study, Column Adsorption and Mechanism Investigation

Xingang Bai[a], Luyang Xing[b], Ning Liu[a]*, Xinyu Wang[b], Nana Ma[b], Kexin Huang[a], Dapeng Wu[a,b*], Mengmeng Yin[a], Kai Jiang[a]*

[a] *School of Environment, Henan Normal University, Key Laboratory for Yellow River and Huai River Water Environmental and Pollution Control, Ministry of Education, Henan Key Laboratory for Environmental Pollution Control, Xinxiang, Henan 453007, PR China*

[b] *School of Chemistry and Chemical Engineering, Henan Normal University, Xinxiang, Henan 453007, P.R. China*

Corresponding author Prof. Kai Jiang jiangkai6898@126.com

Corresponding author Dr. Dapeng Wu dapengwu@htu.edu.cn

Corresponding author Dr. Ning Liu lnywzq@163.com

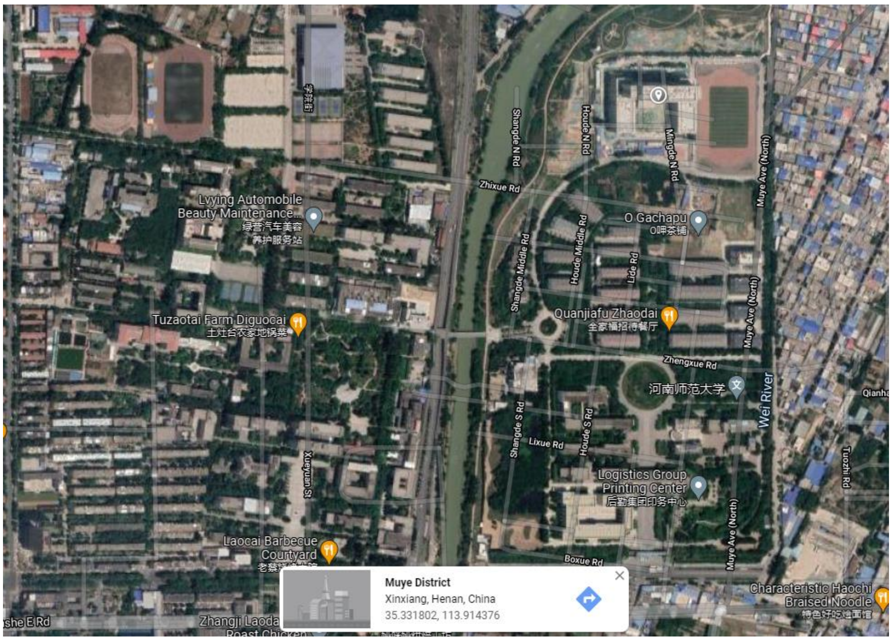

Figure S1 Location of the site where the HS biomass is obtained. (The map is downloaded from an open source website: https://www.google.com/maps/)

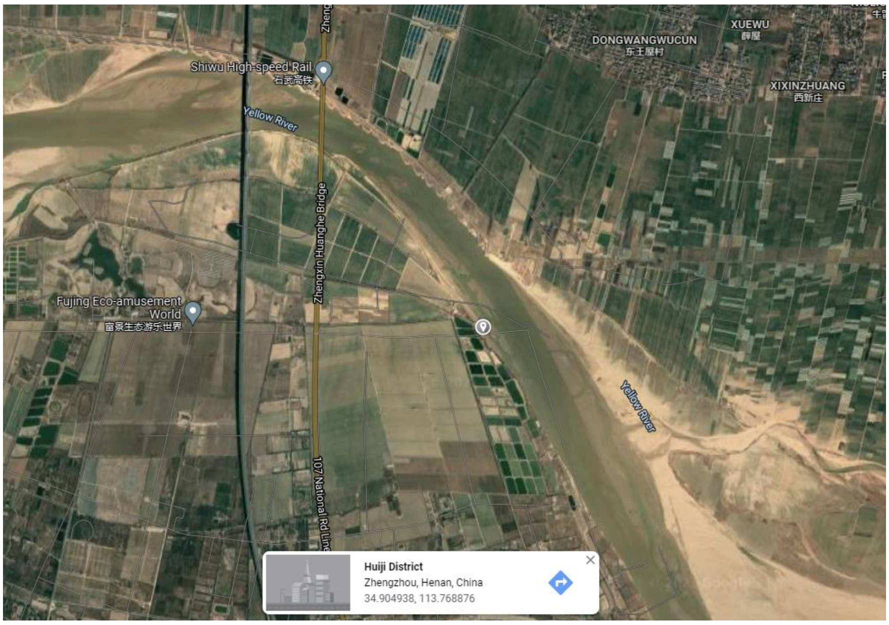

Figure S2 Location of the site where the Yellow river water is fetched. (The map is downloaded from an open source website: https://www.google.com/maps/)

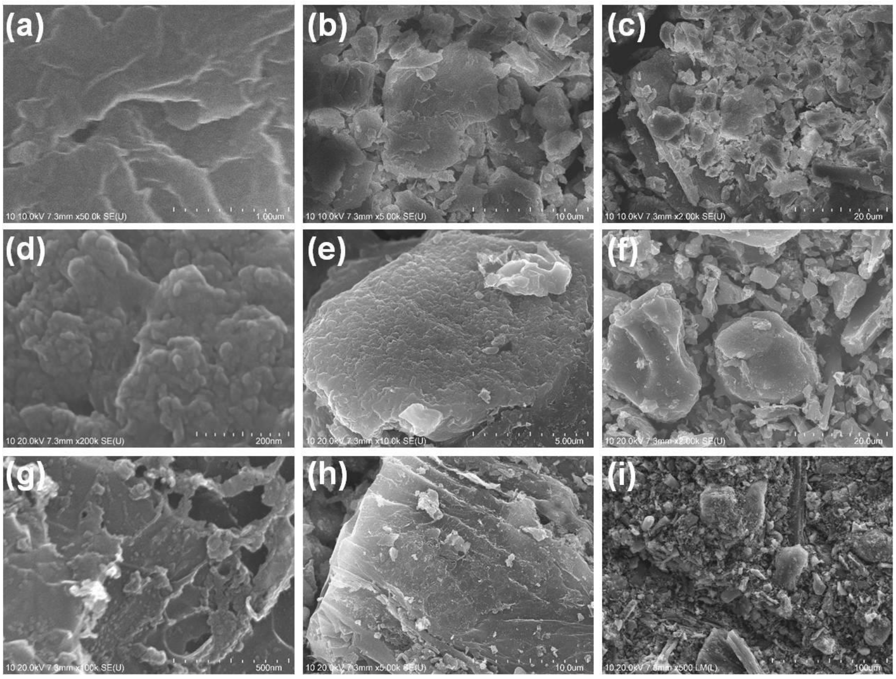

Figure S3 SEM images of HS, HSC-N_2, HSC-M

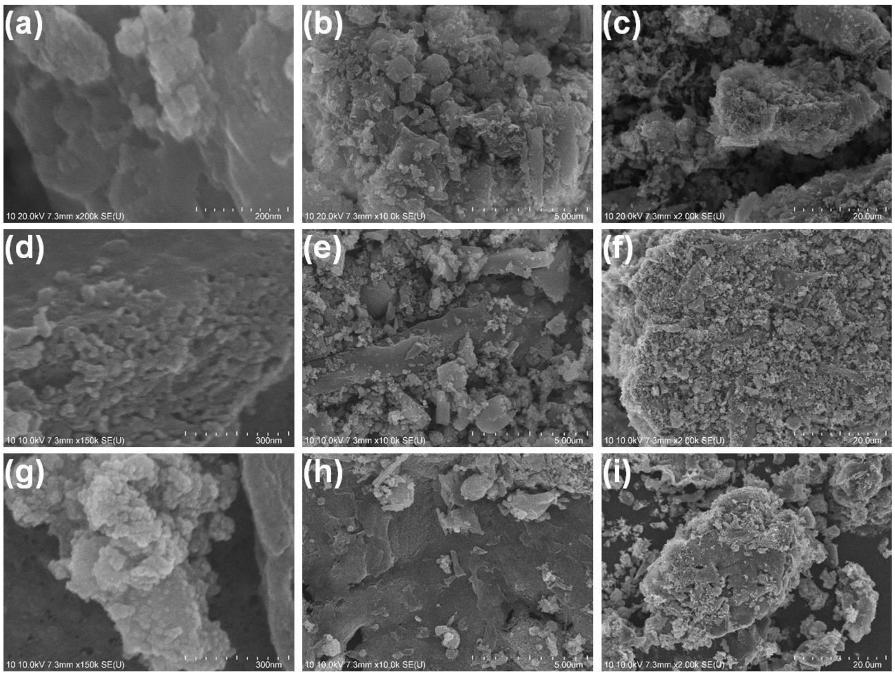

Figure S4 SEM images of HSC-MA-1, HSC-MA-2, HSC-MA-3

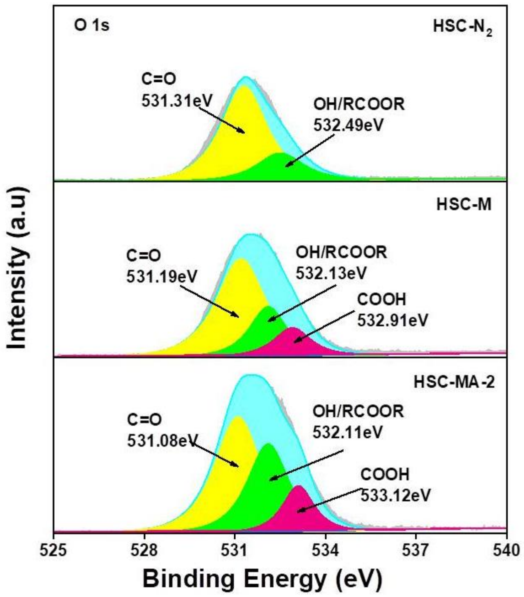

Figure S5 detailed XPS analysis on the C_{1s} of the biochars

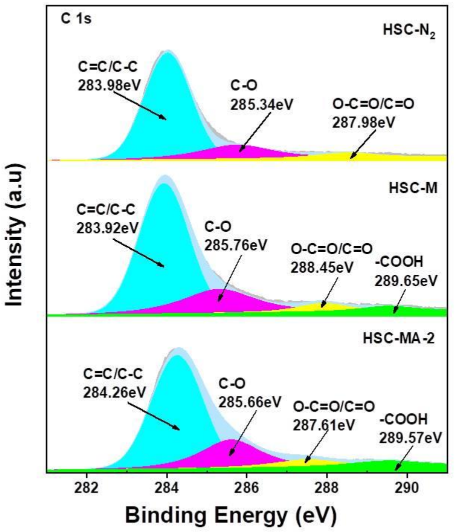

Figure S6 detailed XPS analysis on the O_{1s} of the biochars

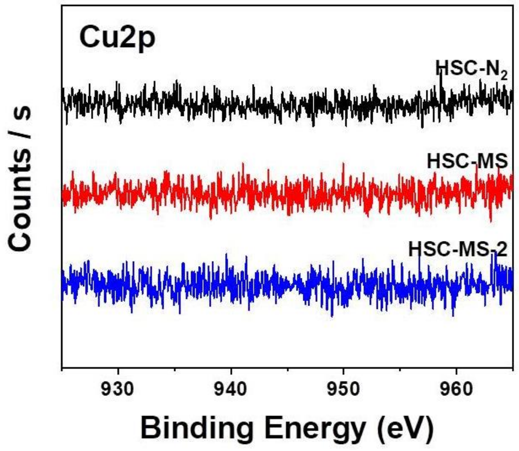

Figure S7 XPS on the Cu_{2p} of the biochar before adsorption

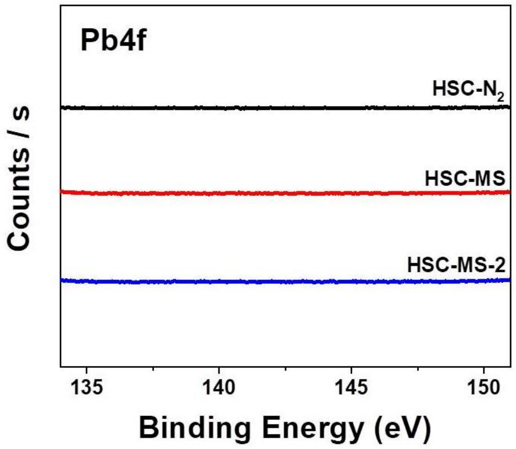

Figure S8 XPS on the Pb_{4f} of the biochar before adsorption
